# Bystanders and Close Ones in Unsuccessful Resuscitation: An Overlooked Population in Out-of-Hospital Cardiac Arrest

**DOI:** 10.3390/nursrep16100354

**Published:** 2026-10-08

**Authors:** Guglielmo Imbriaco, Nino Fijačko, Sergio Cazorla-Calderón, Kristin Alm-Kruse, Marco Mion

**Affiliations:** 1 118 Emilia Est Emergency Medical Communication Centre, Maggiore Hospital, 40133 Bologna, Italy; 2 Faculty of Health Sciences, University of Ljubljana, 1000 Ljubljana, Slovenia; 3 Institute for Research and Innovation in Life Sciences and Health in Central Catalonia, University of Vic—Central University of Catalonia, 08500 Vic, Spain; 4 Division of Prehospital Services, Oslo University Hospital, 0424 Oslo, Norway; 5 Department of Research and Development, Division of Emergencies and Critical Care, Oslo University, 0424 Oslo, Norway; 6 Essex Cardiothoracic Centre, Mid and South Essex NHS Foundation Trust, Basildon SS16 5NL, UK; 7 Medical Technology Research Centre (MTRC), Anglia Ruskin School of Medicine, Chelmsford CM1 1SQ, UK

**Keywords:** bystanders, cardiopulmonary resuscitation, emergency nursing, out-of-hospital cardiac arrest, psychological support

## Abstract

Bystanders, lay rescuers, and close ones involved in out-of-hospital cardiac arrest resuscitation attempts that end in death represent an often-overlooked population within emergency care. As community-centered resuscitation systems increasingly promote dispatcher-assisted cardiopulmonary resuscitation (CPR), community first responder programs, and public CPR training, more individuals are becoming directly involved in resuscitation attempts, many of which are unsuccessful. Such experiences can be associated with emotional distress, intrusive memories, guilt, and post-traumatic stress symptoms. The impact may differ according to the person’s relationship with the patient and their involvement in the resuscitation. This perspective paper discusses the psychological impact of unsuccessful resuscitation and considers the role of emergency nurses in providing support across dispatch, prehospital, and hospital settings. Approaches such as supportive follow-up contact and referral for psychological support, as distinct from formal psychological debriefing protocols, could help address some of these needs, although current evidence on their effectiveness remains preliminary. Greater recognition of people involved in unsuccessful resuscitation and their support needs may represent a further step in the development of community-centered resuscitation systems.

## 1. Introduction

Over the past decade, resuscitation systems have increasingly adopted a community-centered approach to out-of-hospital cardiac arrest (OHCA). International resuscitation frameworks, including those developed by the European Resuscitation Council and the American Heart Association, place strong emphasis on strengthening the earliest links in the chain of survival, particularly by improving recognition of cardiac arrest and rapid initiation of bystander cardiopulmonary resuscitation (CPR) [1,2]. Dispatcher-assisted CPR (DA-CPR) has become a central part of this strategy, enabling emergency medical dispatchers to guide callers, substantially increasing the proportion of victims receiving immediate resuscitation before emergency medical services (EMS) arrival [3]. In Europe, about 70% of OHCA occur in private residences [4], where family members are often the first witnesses of collapse, making close ones and lay rescuers critical early links in the resuscitation chain.

Expanding bystander participation is widely recognized as one of the most effective ways to improve survival after OHCA [5]. However, despite continuous improvements in community response and resuscitation systems, survival remains limited, with survival to hospital discharge rates below 10% in most developed healthcare systems [5,6]. In Europe, resuscitation is started or continued by EMS in approximately 56 cases per 100,000 inhabitants per year, bystander CPR is performed in about 60% of cases not witnessed by EMS, and only 7.5% of patients survive [6]. More than nine out of ten resuscitation attempts therefore end in death, and about two-thirds of patients die on scene [6]. Emergency medical dispatchers also provided telephone CPR instructions in about half of all cases in an earlier European registry study [7]. Strengthening early response also increases the number of citizens exposed to potentially traumatic events with fatal outcomes.

A recent scoping review mapped existing support interventions for OHCA bystanders but did not differentiate by patient outcome and found that none of the included interventions had been evaluated for effectiveness [8]. A separate systematic review and qualitative meta-synthesis characterized the care needs of families experiencing sudden cardiac arrest [9]. This perspective is not meant to duplicate that evidence synthesis. Instead, it focuses on individuals involved in resuscitation attempts that end in death and proposes a preliminary role for emergency nurses in coordinating support across the dispatch, prehospital, and hospital continuum, an aspect that neither prior work addresses as an organizing focus. The perspective draws on a targeted, non-systematic review of literature published mainly from 2015 onwards, supplemented by selected earlier key references, identified through PubMed and manual reference screening, prioritizing studies on psychological outcomes in bystanders, lay rescuers, or family members after OHCA. Findings should be read as illustrative of an emerging body of evidence, not as a comprehensive or systematically weighted synthesis.

In many systems, emergency nurses work across the resuscitation continuum, from emergency medical communication centers to hospital-based care, although the professional composition of dispatch services varies considerably between countries. Here, “emergency nurses” refers to registered nurses working in emergency medical communication centers, prehospital services, or emergency departments, whatever their local title. They encounter bystanders and close ones at several points: during the emergency call, in transit, and in the emergency department immediately after resuscitation attempts. Despite this central role, current emergency nursing protocols, curricula, and resuscitation guidelines rarely address how to systematically assess, support, or follow up with this population. This represents a significant gap in person-centered emergency care and an opportunity for nursing leadership at both clinical and policy levels.

## 2. The Psychological Impact of Unsuccessful Resuscitation

The psychological consequences on lay rescuers, community first responders, and relatives participating in resuscitation attempts have been explored in several studies [10,11,12,13]. Qualitative and quantitative research indicate that individuals who perform CPR may experience emotional stress, intrusive memories, or doubts about their actions in the aftermath of the event [12,13,14]. For example, qualitative interviews with lay rescuers describe a mixture of altruistic motivation and emotional strain, particularly when the experience of real-life resuscitation differs from expectations formed during training [12,14]. Studies from several countries document that some rescuers report worries about having performed the maneuvers incorrectly or having unintentionally harmed the victim [12,13,14].

The psychological burden may be even more pronounced when relatives witness or participate in unsuccessful resuscitation attempts involving a loved one [15]. Family members who witness a loved one’s cardiac arrest describe vivid, intrusive memories and lasting psychological consequences [16]. However, being present does not in itself appear to worsen outcomes: in a randomized trial in the out-of-hospital setting, relatives who were offered the opportunity to witness resuscitation reported fewer symptoms of post-traumatic stress disorder (PTSD) at 90 days than those who were not [17]. This suggests that the relevant focus is support after the event.

Qualitative and quantitative studies have shown that exposure to cardiac arrest events may lead to symptoms consistent with acute stress reactions or PTSD in a subset of participants [13,14,15,16]. Resuscitation attempts can represent psychologically traumatic events requiring appropriate evaluation and follow-up strategies. For example, among lay rescuers alerted by text message, 13% reported a severe short-term psychological impact, although none had moderate or severe PTSD-related symptoms 4–6 weeks later [13]. The number of people exposed to fatal resuscitation attempts is not recorded in cardiac arrest registries, and data on the frequency of psychological distress or unmet support needs are scarce, heterogeneous, and rarely stratified by patient outcome or by the person’s relationship with the patient. This lack of evidence is itself an important gap in the current literature.

Current evidence introduces a renewed focus on survivors and “co-survivors”, a concept acknowledging that the consequences of cardiac arrest extend beyond the patient [9,18]. Family members, witnesses, and individuals involved in the emergency response may experience long-lasting emotional and psychological effects following the event [15,16]. However, within this broader category, bystanders and lay rescuers who are not relatives of the victim, yet who actively participate in resuscitation, represent a distinct subgroup with different psychological needs than family members experiencing bereavement [10,11,12,13,14]. Current guidelines also encourage structured initiatives to support survivors and their relatives during the recovery process, including follow-up programs, information sharing, and community engagement activities designed to improve long-term well-being after cardiac arrest [1,19], but these initiatives are designed mainly around patients who survived.

The term “co-survivor” is not applied consistently across this literature. Several studies define co-survivorship specifically as the experience of family members of patients who survived to hospital discharge, treating bereaved family members as a separate category rather than a subset of co-survivors [9,15]. Other sources use the term more broadly to include bereaved relatives. This leaves at least two groups poorly represented in the existing evidence: bereaved family members, whose inclusion under “co-survivorship” varies by study, and non-family bystanders or lay rescuers involved in a fatal resuscitation, who are captured by neither the “co-survivor” nor the “bereaved family” framework as conventionally defined. The remainder of this article addresses both bereaved co-survivors and non-family bystanders of unsuccessful resuscitation. In informal discussions with people with lived experience of cardiac arrest consulted during the preparation of this paper, views on the term “co-survivor” itself were mixed: some found it validating, while others felt it did not fit their experience, particularly after an unsuccessful resuscitation, and preferred language that did not imply victimhood.

## 3. An Overlooked Population in Contemporary Resuscitation Systems

For the purposes of this paper, we define the target population as: (a) family members or close ones who witnessed and/or participated in the resuscitation of someone they knew; (b) lay rescuers with no prior relationship to the patient, including community first responders [20] alerted through smartphone-based systems, who performed CPR and/or used an automated external defibrillator (AED); and (c) callers who received dispatcher-assisted CPR instructions by phone, regardless of whether they physically performed compressions. On-duty (professional) first responders, such as firefighters or police officers acting in an occupational capacity, are excluded, as their exposure and support needs differ and warrant separate consideration. As outlined above, groups (a) and (b) are not consistently captured by the “co-survivor” or “bereaved family” frameworks used in prior evidence syntheses [8,9,15]; this perspective addresses that gap. We use “unsuccessful resuscitation” to refer to those attempts in which the patient dies, either on scene after termination of resuscitation or later in hospital. The terms used throughout this paper are defined in Table 1.

This population includes people who are directly involved in resuscitation attempts that do not result in survival. In many cases, they are not passive witnesses but active participants in resuscitation, delivering chest compressions, operating an AED, or assisting with other life-saving interventions. Although these groups differ in their relationship to the patient and the nature of their involvement, they share exposure to a potentially traumatic event and may experience emotional distress, uncertainty, guilt, or psychological burden in the aftermath of the resuscitation attempt [12,18]. Despite these potential consequences, the experiences and support needs of these individuals remain poorly recognized within contemporary resuscitation systems.

Public awareness campaigns, school-based CPR education programs, smartphone-activated community first responder systems, and DA-CPR protocols are valuable initiatives that encourage citizens to intervene. However, because most OHCAs remain fatal, many citizens who respond to these emergencies will face an unsuccessful outcome. Ensuring that these individuals are appropriately supported should therefore represent an important, yet underdeveloped, component of community-centered resuscitation systems. Recognizing this group of bystanders, lay rescuers, and close ones as a distinct population becomes particularly relevant in modern resuscitation systems that actively promote citizen engagement.

Some groups within this population warrant particular attention. As school-based CPR education expands, children and adolescents may witness or take part in resuscitation attempts, often of a family member; follow-up with minors should involve parents or guardians and use age-appropriate communication and referral pathways. Language and cultural background may also shape how distress is expressed and which forms of support are acceptable, and preferences for support interventions differ among racially and ethnically diverse family members [21]. Follow-up contact should include access to interpreters and culturally adapted materials.

## 4. Emergency Nursing’s Critical Role in Post-Resuscitation Support

Understanding the psychological trajectory of bystanders and close ones is essential. Emergency nurses have an important role in the continuum of cardiac arrest care, especially in supporting relatives and bystanders. From emergency medical communication centers to hospital-based care, nurses can provide structured support and lead the implementation of family-centered care models, ensuring that communication is clear and empathetic, mitigating the initial trauma of the event [9]. Supportive follow-up differs from structured psychological debriefing protocols, which involve organized processing of the traumatic event, typically in a single session. Evidence on single-session debriefing after traumatic events is mixed, and some studies report limited or even adverse effects when it is mandatory rather than offered as an option [22,23]. This paper refers to the low-intensity, opt-in kind of contact, not structured debriefing.

Emergency medical dispatchers, including nurses in emergency medical communication centers, hold a particular position in the resuscitation process: they guide callers through the resuscitation attempt and remain connected with them throughout the critical early minutes of the emergency. In some cases, they are the first and the last professionals to interact with the caller before EMS arrival. This relationship creates an opportunity to implement simple supportive follow-up contact strategies to acknowledge the caller’s effort, clarify uncertainties, and provide reassurance [24]. Even brief interventions, such as validating the bystander’s contribution and explaining that many cardiac arrests are not survivable despite optimal efforts, may help mitigate feelings of guilt or distress.

Similar approaches have been explored in programs involving community first responders, where structured feedback and psychological support have been suggested as mechanisms to reduce post-event stress [25]. Although evidence regarding the effectiveness of these interventions remains limited, available studies suggest that many lay rescuers value structured feedback, opportunities to ask questions, and acknowledgment of their contribution following the event [26]. In Europe, the need for structured follow-up programs for lay rescuers has been recognized, with proposals for standardized post-event support models that include debriefing and optional psychological support [8,27]. In the United Kingdom, a recent pilot service offering on-scene debrief, registration for support via a mobile app and website, and follow-up by a Patient Liaison Officer provides an alternative example of how this could be implemented [28]. Such models illustrate that systematic follow-up may be feasible within existing emergency care frameworks, although a recent scoping review found that none of the currently available bystander support interventions have been formally evaluated for effectiveness [8], and effectiveness data from the UK pilot service are not yet available [28], so this evidence remains preliminary.

Post-event support may be managed by emergency medical dispatchers, paramedics, emergency nurses, physicians, or multidisciplinary teams, depending on local organization. Beyond the acknowledgment described above, early contact with bystanders and close ones following an unsuccessful resuscitation is also an opportunity to identify individuals who may benefit from additional psychological support. These interventions do not require complex resources. Self-help resources, short follow-up calls, and escalation pathways for psychological support could represent feasible and inexpensive first steps within many emergency care systems [8,26,28].

Many of these interactions take place at a specific, clinically recognizable point in the pathway: the decision to terminate resuscitation on scene. This is the moment when the outcome becomes clear to those present, and it is a natural anchor for acknowledging the effort of bystanders, communicating with family members, and offering later support. In practice, these contact points could follow a simple sequence: brief acknowledgment by the emergency medical dispatcher once resuscitation instructions end, clear and compassionate communication of the outcome on scene to family members, together with a point of contact for later support offered to everyone involved: a short supportive follow-up call or message within the first few days for those who have agreed to be contacted. Making this sequence explicit is a first step toward a more implementable model of support. Those consulted also emphasized that acute shock can make spoken information hard to absorb, and suggested that simple, written or visual materials, such as a short summary of what to expect next, be offered alongside verbal communication.

The content of this contact should reflect differences between groups. For family members and close ones, exposure to the resuscitation attempt overlaps with bereavement; support should therefore address grief as well as the traumatic experience, for example through clear information about the death, the opportunity to ask what happened, and links to bereavement services [9,15,16]. Peer support from other bereaved co-survivors, where accessible through patient and family organizations, was also valued by those consulted. For non-family bystanders and callers, concerns more often relate to the event itself, the actions they performed, and feelings of responsibility for the outcome [12,13,14]; acknowledgment of their contribution, factual feedback on the resuscitation effort within the limits of confidentiality, and, where they already know the outcome, reassurance that the death was not caused by their actions are likely to be most relevant [26]. Many non-family bystanders who assist a stranger never learn the outcome of the resuscitation at all, which follow-up models need to take into account when deciding what, if anything, to offer this group. Some elements of this approach are supported by current evidence: lay rescuers value the opportunity to talk about the event and receive feedback [26], offering relatives the option to be present during resuscitation does not appear to be harmful [17], and mandatory single-session debriefing is not recommended [22,23]. Preliminary findings from the RescQ pilot study suggest that a digital platform with optional escalation to personalized support is feasible and acceptable to bystanders after OHCA, although its effectiveness remains uncertain [28]. Other approaches, including structured follow-up calls and nurse-coordinated pathways, require further evaluation [8].

Structured follow-up can also have unintended effects. Bystanders or family members may perceive unsolicited contact as intrusive if the timing or content is poorly judged, a concern echoed in postvention literature on sudden bereavement [29]. Debriefing that is mandatory or too clinical in tone risks pathologizing normal grief or stress reactions [22,23]. Follow-up programs need adequately trained staff and clear escalation pathways; without consistent implementation, they risk offering false reassurance rather than real support. These programs should be opt-in, flexible in timing, and evaluated on an ongoing basis for both benefit and harm. Support needs described by those consulted did not always arise immediately: for some they resurfaced weeks or months later, including around anniversaries of the event, so follow-up pathways should keep a later door open rather than closing after an initial contact, while remaining mindful of the timing of inquests or funerals.

Implementing follow-up contact may also raise legal and ethical issues that differ between jurisdictions. Contact details obtained during an emergency call are collected to manage the emergency, not to provide later support; in the European Union, reusing them for follow-up requires a lawful basis under the General Data Protection Regulation (Article 6) and, because health-related information is involved, a condition for processing special categories of data (Article 9). A practical approach is to ask for consent at a defined moment, for example at the end of the call or on scene once resuscitation has ended, using a brief, standardized script, and to document it. Follow-up with non-family bystanders and callers should focus on their own experience and should not disclose clinical information about the patient, in keeping with professional confidentiality. Local policies should also specify who may access call recordings and contact data for this purpose and for how long they are retained. Addressing these issues explicitly is a precondition for implementation and partly explains why existing programs differ across European systems.

## 5. Strengthening Community Resuscitation Systems

As resuscitation science moves toward a systems-based model, the psychological needs of those who witness, respond to, or are otherwise affected by a cardiac arrest should be considered part of person-centered emergency care. Emergency medical dispatchers, bystanders, survivors, and families all contribute to the chain of survival and share in the experience of these critical events. As noted above, emergency nurses are not the only professionals who can provide this support, but their continuous presence across dispatch, prehospital, and hospital settings puts them in a good position to coordinate and integrate these efforts throughout the resuscitation continuum. Those consulted also described confusion when several agencies were involved with no single point of contact, reinforcing the value of a coordinating role of this kind.

Taking on this coordinating role requires specific competencies. Most initial support falls within routine emergency nursing practice: acknowledging the person’s contribution, communicating clearly and compassionately with bereaved relatives and others exposed to a potentially traumatic event, providing basic information on common stress reactions, and signposting available support. Coordinating follow-up requires additional training in recognizing signs of persistent or severe distress, applying agreed escalation criteria, and activating referral pathways, as well as knowledge of the legal and confidentiality issues described. Assessment and treatment of suspected acute stress disorder, PTSD, or complicated grief remain the responsibility of mental health professionals, and nurse-coordinated pathways should include clear and timely routes to these services. These competencies could be incorporated into existing emergency nursing and dispatcher training, although their content and effectiveness have not yet been evaluated.

Future research should better characterize the experiences and needs of bystanders and close ones involved in unsuccessful resuscitation attempts, including the prevalence and severity of psychological distress, and should test whether supportive follow-up improves their outcomes. Agreeing on a core outcome set, including validated measures of psychological distress and common assessment time points, would make findings from different systems comparable. Qualitative research may provide valuable insights into the emotional trajectories of relatives and community first responders after cardiac arrest events. Feasibility studies and pragmatic designs such as stepped-wedge implementation trials are well suited to services introducing follow-up programs, and linkage with existing OHCA registries would allow outcomes to be analyzed by patient outcome and type of involvement. Such knowledge could inform the development of evidence-based support strategies integrated into the broader resuscitation system.

This perspective has several limitations. It draws on a targeted, non-systematic review of the literature, based on a single database (PubMed) supplemented by manual reference screening; the quality of the included studies was not formally appraised, and only English-language publications were considered. The available evidence comes mainly from high-income countries, and data from low- and middle-income countries, where resuscitation systems and support resources differ, are largely lacking. Input from people with lived experience was informal and limited to three individuals; future support interventions should be formally co-designed with bystanders and bereaved relatives.

Developing structured support pathways for bystanders, lay rescuers, community first responders, and close ones involved in unsuccessful resuscitation attempts acknowledges that the impact of cardiac arrest extends beyond survivors and their families. This is a logical next step for community-centered resuscitation systems, and one in which emergency nursing can take the lead.

## Figures and Tables

**Table 1:** Definitions of terms used for people involved in out-of-hospital cardiac arrest.

Term	Definition
Bystander	Any person present at the OHCA scene or alerted but not dispatched as part of an organized response.
Close one	Family member, partner, friend, or other person with a prior personal relationship with the patient.
Lay rescuer	Bystander without a professional duty to respond who performs CPR and/or uses an AED.
Community first responder	Volunteer without a professional duty to respond who is alerted to a nearby OHCA, either through a smartphone application or text message without being officially dispatched (“volunteer community responder”) or as part of an organized community first responder scheme activated by emergency medical services.
Co-survivor	Family members and close ones of cardiac arrest patients; in several studies restricted to relatives of patients who survive to hospital discharge, so its scope varies.

## Data Availability

No new data were created or analyzed in this study. Data sharing is not applicable to this article.

## Public Involvement Statement

Informal input was sought from three people with lived or professional experience of out-of-hospital cardiac arrest, contacted through survivor and family support organizations in Sweden and the United Kingdom. Their perspectives informed several revisions to this manuscript. This input was collected informally and was not treated as research data.

## Guidelines and Standards Statement

Not applicable; this perspective does not report primary research, a systematic review, or a study type requiring a reporting guideline.

## Abbreviations

The following abbreviations are used in this manuscript:

AED	Automated External Defibrillator
CPR	Cardiopulmonary Resuscitation
DA-CPR	Dispatcher-Assisted Cardiopulmonary Resuscitation
EMS	Emergency Medical Services
OHCA	Out-of-Hospital Cardiac Arrest
PTSD	Post-Traumatic Stress Disorder

## References

[1] Semeraro F., Schnaubelt S., Olasveengen T.M., Bignami E.G., Böttiger B.W., Fijačko N., Gamberini L., Hansen C.M., Lockey A., Metelmann B. (2025). European Resuscitation Council Guidelines 2025 System Saving Lives. Resuscitation.

[2] Dezfulian C., Cabañas J.G., Buckley J.R., Cash R.E., Crowe R.P., Drennan I.R., Mahgoub M., Mannarino C.N., May T., Salcido D.D. (2025). Part 4: Systems of Care: 2025 American Heart Association Guidelines for Cardiopulmonary Resuscitation and Emergency Cardiovascular Care. Circulation.

[3] Kurz M.C., Bobrow B.J., Buckingham J., Cabanas J.G., Eisenberg M., Fromm P., Panczyk M.J., Rea T., Seaman K., Vaillancourt C. (2020). Telecommunicator Cardiopulmonary Resuscitation: A Policy Statement From the American Heart Association. Circulation.

[4] Baldi E., Wnent J., Caputo M.L., Haywood K.L., Lilja G., Masterson S., Nehme Z., Perkins G.D., Rosell-Ortiz F., Strömsöe A. (2025). European Resuscitation Council Guidelines 2025 Epidemiology in Resuscitation. Resuscitation.

[5] Simmons K.M., McIsaac S.M., Ohle R. (2023). Impact of Community-Based Interventions on Out-of-Hospital Cardiac Arrest Outcomes: A Systematic Review and Meta-Analysis. Sci. Rep..

[6] Gräsner J.-T., Wnent J., Lefering R., Herlitz J., Masterson S., Maurer H., Perkins G.D., Ortiz F.R., Tjelmeland I.B., Kamishi D. (2026). European Registry of Cardiac Arrest Study THREE (EuReCa-THREE): EMS Response Time Influence on Outcome in Europe. Resuscitation.

[7] Gräsner J.T., Wnent J., Herlitz J., Perkins G.D., Lefering R., Tjelmeland I., Koster R.W., Masterson S., Rossell-Ortiz F., Maurer H. (2020). Survival after Out-of-Hospital Cardiac Arrest in Europe—Results of the EuReCa TWO Study. Resuscitation.

[8] Sajjad U., Mion M., Khan S., Lindner T.W., Pareek N., Keeble T.R., Joshi V.L. (2026). A Scoping Review of Support Interventions for Bystanders Involved in Out-of-Hospital Cardiac Arrest. Resuscitation.

[9] Douma M.J., Myhre C., Ali S., Graham T.A., Ruether K., Brindley P.G., Dainty K.N., Smith K.E., Montgomery C.L., Dennet L. (2023). What Are the Care Needs of Families Experiencing Sudden Cardiac Arrest? A Survivor- and Family-Performed Systematic Review, Qualitative Meta-Synthesis, and Clinical Practice Recommendations. J. Emerg. Nurs..

[10] Simpson R.F.G., Mion M., Sajjad U., Swindell P., Keeble T.R. (2024). Highlighting Reported Psychological Issues of Co-Survivors Post-Delivery of CPR in Out-of-Hospital Cardiac Arrest: A Qualitative Study. Resuscitation.

[11] Ganter J., Ruf A.-C., Bushuven S., Nowotny-Behrens U., Müller M.P., Busch H.-J. (2025). Psychological Impact on First Responders Dispatched to Out-of-Hospital Cardiac Arrest via Smartphone Alerting System: A Longitudinal Survey-Based Study. Resusc. Plus.

[12] Li N., Shen C., Yang X., Wang R., Gu L.Q., Zhao W., Chu Z.P. (2024). The Real Experience of Lay Responders Performing Cardiopulmonary Resuscitation: A Synthesis of Qualitative Evidence. Public Health Rev..

[13] Zijlstra J.A., Beesems S.G., De Haan R.J., Koster R.W. (2015). Psychological Impact on Dispatched Local Lay Rescuers Performing Bystander Cardiopulmonary Resuscitation. Resuscitation.

[14] Mathiesen W.T., Bjørshol C.A., Braut G.S., Søreide E. (2016). Reactions and Coping Strategies in Lay Rescuers Who Have Provided CPR to Out-of-Hospital Cardiac Arrest Victims: A Qualitative Study. BMJ Open.

[15] Rojas D.A., DeForge C.E., Abukhadra S.L., Farrell L., George M., Agarwal S. (2023). Family Experiences and Health Outcomes Following a Loved Ones’ Hospital Discharge or Death after Cardiac Arrest: A Scoping Review. Resusc. Plus.

[16] Dainty K.N., Amog K., Agarwal S., Seaton M.B. (2025). “I Can’t Unsee Him Lying in My Kitchen”: Understanding the Trauma of Family Members Who Witness a Loved One’s Cardiac Arrest. Resusc. Plus.

[17] Jabre P., Belpomme V., Azoulay E., Jacob L., Bertrand L., Lapostolle F., Tazarourte K., Bouilleau G., Pinaud V., Broche C. (2013). Family Presence during Cardiopulmonary Resuscitation. N. Engl. J. Med..

[18] Kuehn B.M. (2024). Close Family Members or Cosurvivors of Cardiac Arrest Patients Need Support. Circulation.

[19] Gamberini L., Haywood K.L., Schnaubelt S., Thyssens M., Semeraro F., Monsieurs K.G., Agarwal S., Blek-Vehkaluoto M., Cerchiari E., Israelsson J. (2025). Organisations Supporting Cardiac Arrest Survivors: An Exploratory Survey of Organisational Structures and Activities. Resusc. Plus.

[20] Metelmann C., Metelmann B., Müller M.P., Scquizzato T., Baldi E., Barry T., Böttiger B.W., Busch H.J., Caputo M.L., Cheskes S. (2025). Defining the Terminology of First Responders Alerted for Out-of-Hospital Cardiac Arrest by Medical Dispatch Centres: An International Consensus Study on Nomenclature. Resusc. Plus.

[21] Agarwal S., Tincher I.M., Abukhadra S.L., Rojas D.A., DeForge C.E., Marchionda C., Wylie J., Chap J. (2024). Prioritizing Intervention Preferences to Potentially Reduce Caregiver Burden in Racially and Ethnically Diverse Close Family Members of Cardiac Arrest Survivors. Resuscitation.

[22] Ancarani F., Garijo Añaños P., Gutiérrez B., Pérez-Nievas J., Vicente-Rodríguez G., Gimeno Marco F. (2025). The Effectiveness of Debriefing on the Mental Health of Rescue Teams: A Systematic Review. Int. J. Environ. Res. Public Health.

[23] Rose S.C., Bisson J., Churchill R., Wessely S. (2002). Psychological Debriefing for Preventing Post Traumatic Stress Disorder (PTSD). Cochrane Database Syst. Rev..

[24] Imbriaco G., Ramacciati N. (2024). Optimizing Dispatcher-Bystander Dyadic Collaboration in Emergency Medical Communication to Improve Cardiac Arrest Response. Clin. Exp. Emerg. Med..

[25] Allert C., Nilsson B., Svensson A., Andersson E.K. (2024). Voluntary First Responders’ Experiences of Being Dispatched to Suspected Out-of-Hospital Cardiac Arrest in Rural Areas: An Interview Study. BMC Cardiovasc. Disord..

[26] Møller T.P., Hansen C.M., Fjordholt M., Pedersen B.D., Østergaard D., Lippert F.K. (2014). Debriefing Bystanders of Out-of-Hospital Cardiac Arrest Is Valuable. Resuscitation.

[27] Schnaubelt S., Orlob S., Veigl C., Sulzgruber P., Krammel M., Lauridsen K.G., Greif R. (2023). Out of Sight—Out of Mind? The Need for a Professional and Standardized Peri-Mission First Responder Support Model. Resusc. Plus.

[28] Sajjad U., Mion M., Simpson R., Sharpe L., Davis J., Butt H., Roberts-Andreou L., DiFede J., Grier G., Swindell P. (2026). A Stepped-Care Model for Bystander Support after Out-of-Hospital Cardiac Arrest: Findings from the RescQ Pilot [Conference Abstract]. Resuscitation.

[29] Hill N.T.M., Walker R., Andriessen K., Bouras H., Tan S.R., Amaratia P., Woolard A., Strauss P., Perry Y., Lin A. (2022). Reach and Perceived Effectiveness of a Community-Led Active Outreach Postvention Intervention for People Bereaved by Suicide. Front. Public Health.

